# Schmorl's Node Mimicking Spinal Metastatic Disease: A Case Report and Review of the Literature

**DOI:** 10.7759/cureus.70723

**Published:** 2024-10-02

**Authors:** Michael S Schallmo, Katherine D Drexelius, William A Ahrens, Joshua C Patt

**Affiliations:** 1 Orthopedic Surgery, Atrium Health Musculoskeletal Institute, Charlotte, USA; 2 Pathology, Atrium Health Levine Cancer Institute, Charlotte, USA; 3 Orthopedic Surgery, Atrium Health Levine Cancer Institute, Charlotte, USA

**Keywords:** lytic, mimicker, schmorl, spinal metastatic disease, spine lesion

## Abstract

In this report, we present a progressively enlarging, degenerative, intraspongious/intravertebral herniated nucleus pulposus, also referred to as a "Schmorl's node," in a 65-year-old patient with a history of prostate cancer. The patient initially presented to our orthopedic oncology clinic for the evaluation of lytic-appearing lesions involving the L4 and L5 vertebral bodies. He had been diagnosed with prostate cancer approximately four years prior and had been previously treated with prostatectomy. During evaluation for symptoms of neurogenic claudication, computed tomography (CT) demonstrated a hypodense lesion in the L5 vertebral body, which demonstrated mildly increased uptake in the left side of L5 on technetium pyrophosphate nuclear scintigraphy and 18 fluorine fluorodeoxyglucose positron emission tomography-CT scan. CT-guided fine-needle aspiration (FNA) of the lesion was performed and demonstrated no neoplastic findings. He underwent an L4-L5 microscopic unilateral laminotomy with bilateral decompression. However, his neurogenic claudication gradually returned, and he presented to his spine surgeon for further evaluation. Repeat CT of the lumbar spine demonstrated marked interval expansion of the erosive L5 lesion with poorly defined margins as well as a hypodense, erosive lesion in the left side of L4. The patient underwent a repeat FNA, along with a CT-guided core needle biopsy of the lesion at the outside facility which yielded a non-diagnostic specimen. After an extensive discussion with the patient, the decision was ultimately made to proceed with an open biopsy of the L5 lesion with partial L5 corpectomy via left-sided transpedicular approach and L4-S1 decompression and instrumented posterolateral spinal fusion. The primary purpose of the operation was to remove material from the lesion, directly visualize it, and have ample tissue for histopathological analysis. Based on these intraoperative findings and subsequent final histopathologic evaluation, the lesion was definitively diagnosed as a large, aggressive, intraspongious/intravertebral herniated nucleus pulposus. While the differentiation of non-neoplastic conditions, such as a Schmorl's node, from osseous metastatic spine disease can be elusive, it is essential for the appropriate management of patients with a history of malignancy.

## Introduction

Prostate cancer is one the most common cancers to spread to the bone, and over half of patients with prostate cancer may develop metastatic spine disease [1,2]. Prostate cancer may produce sclerotic- or lytic-appearing bone lesions, though lytic is far less common [3-5]. It is important to maintain a very high index of suspicion for metastatic disease in adult patients who present with a new lytic-appearing bone lesion, especially those with a known history of malignancy with a high predilection for osseous metastasis [6]. The astute clinician must also consider the wide array of both neoplastic and non-neoplastic conditions that can mimic metastatic extradural spine disease (Appendices) [1,7-8]. Non-neoplastic conditions, or so-called "mimickers," can include anatomic variants, trauma, degenerative changes, infection, abnormalities related to metabolic conditions, and vascular abnormalities [9,10]. A number of benign and malignant neoplastic conditions can also arise in the spine and require careful consideration during the evaluation of a new spinal lesion. Prostate, breast, thyroid, bladder, lung, and kidney cancer are thought to have the highest incidence of bone metastasis [11]. Therefore, a heightened awareness of the risk of osseous metastasis in these patients is crucial to ensuring this diagnosis is not missed. In this case report, we detail the clinical course of a 65-year-old male with a history of prostate cancer status post prostatectomy who presented with enlarging, destructive-appearing lesions in the vertebral bodies of L4 and L5.

## Case presentation

**Figure 1:**
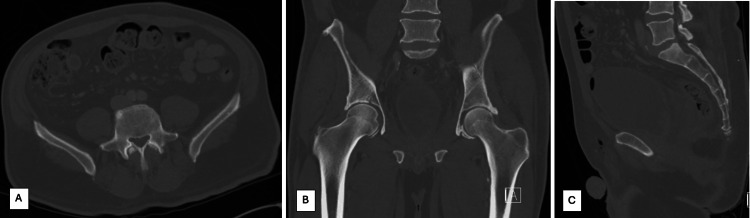
Non-contrast computed tomography of the pelvis performed around the time of the patient's prostatectomy, which did not demonstrate any abnormal osseous lesions. Limited visualization of the L4 vertebral body given the scan had been ordered as a "CT pelvis." Figure 1A demonstrates an axial cut through the superior aspect of the L5 vertebral body, with no lesion evident. Figure 1B demonstrates a coronal cut, allowing for the visualization of the mid-portion of the L5 vertebral body without any osseous lesion identified. Figure 1C demonstrates a sagittal cut, allowing for the visualization of the mid-portion of the L5 vertebral body, also with no lesion noted.

A 65-year-old Caucasian male with a pertinent medical history that includes low-grade prostate adenocarcinoma, mixed urinary incontinence, coronary and peripheral arterial occlusive disease, and multilevel lumbar spondylosis with intractable bilateral neurogenic claudication was referred to orthopedic oncology for the evaluation of lytic-appearing lesions involving the L4 and L5 vertebral bodies. The patient provided verbal consent to be included in this case report. The patient's prostate cancer was diagnosed four years prior and was Gleason score 3+3 (low-grade/well-differentiated; three of 12 positive biopsy regions/cores: left middle base 3%, left lateral base 3%, and left lateral middle 7%). The tumor was American Joint Committee on Cancer stage T2 MX NX, Grade Group 1 (GG1), and the initial total prostate-specific antigen (PSA) was 7.5 ng/mL (16% free PSA) at the time of diagnosis. He subsequently underwent robot-assisted laparoscopic prostatectomy five months after diagnosis; the lesion was confirmed as prostate adenocarcinoma, usual acinar type, organ-confined, and margin-negative. He did not undergo any pharmacotherapy or radiotherapy. His surveillance total PSA tests all remained <0.1 ng/mL following surgery, and there were no documented concerns for new, recurrent, or distant disease. He had a computed tomography (CT) of the pelvis performed around the time of his prostatectomy due to postoperative hematuria, which did not demonstrate any abnormal osseous lesions (Figure 1).

**Figure 2:**
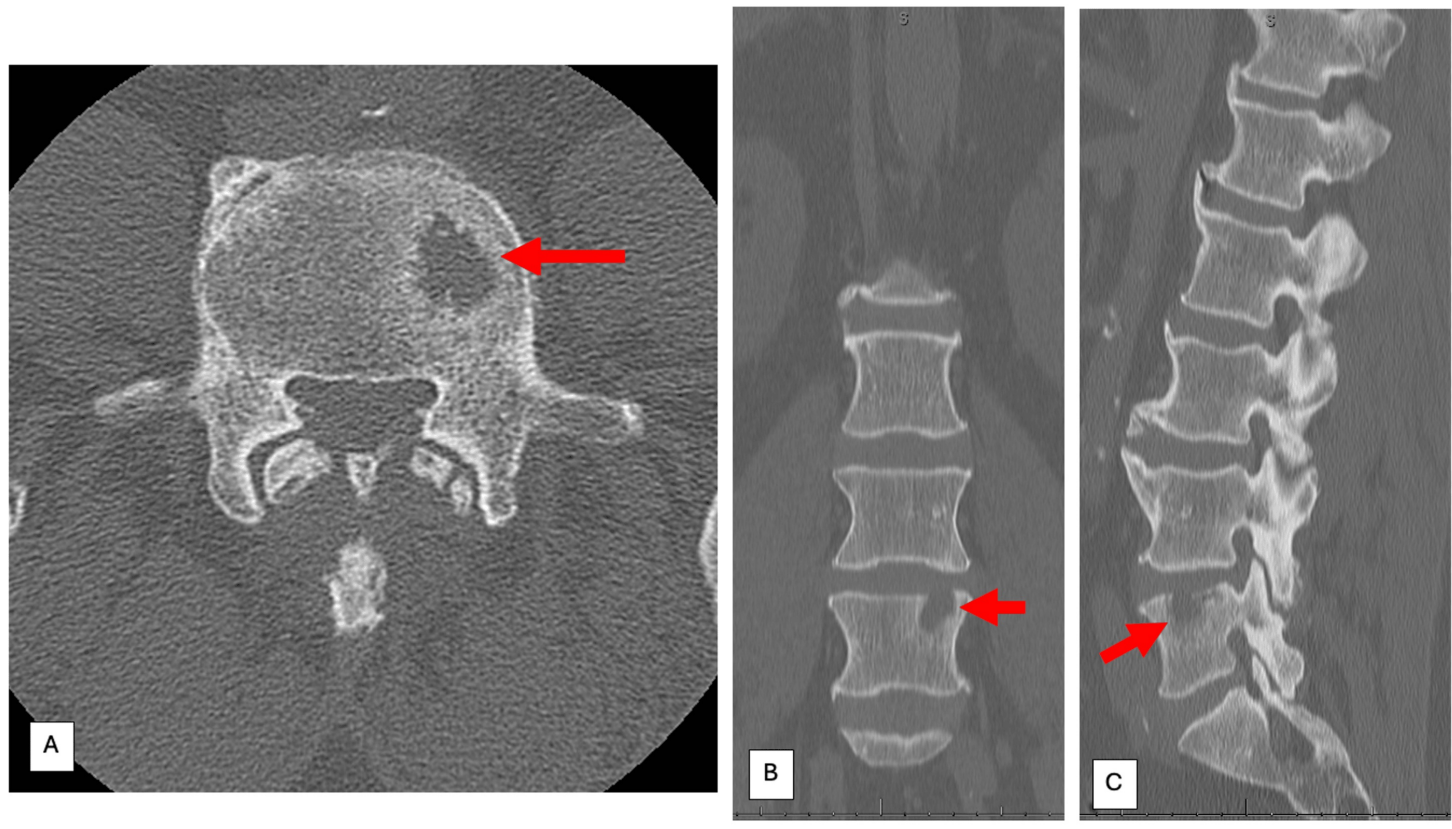
Non-contrast computed tomography of the lumbar spine obtained as part of the initial evaluation for neurogenic claudication, approximately 2.5 years after the patient's diagnosis of prostate cancer and approximately 1.5 years prior to his presentation to our office. This was the earliest image that demonstrated an L5 vertebral body lesion. At that time, the hypodense lesion measured 18.7×12.6×12 mm. Figures 2A, 2B, and 2C demonstrate the L5 vertebral body and associated lesion on axial, coronal, and sagittal cuts, respectively. Red arrows denote the L5 vertebral body lesion on axial, coronal, and sagittal computed tomography imaging.

**Figure 3:**
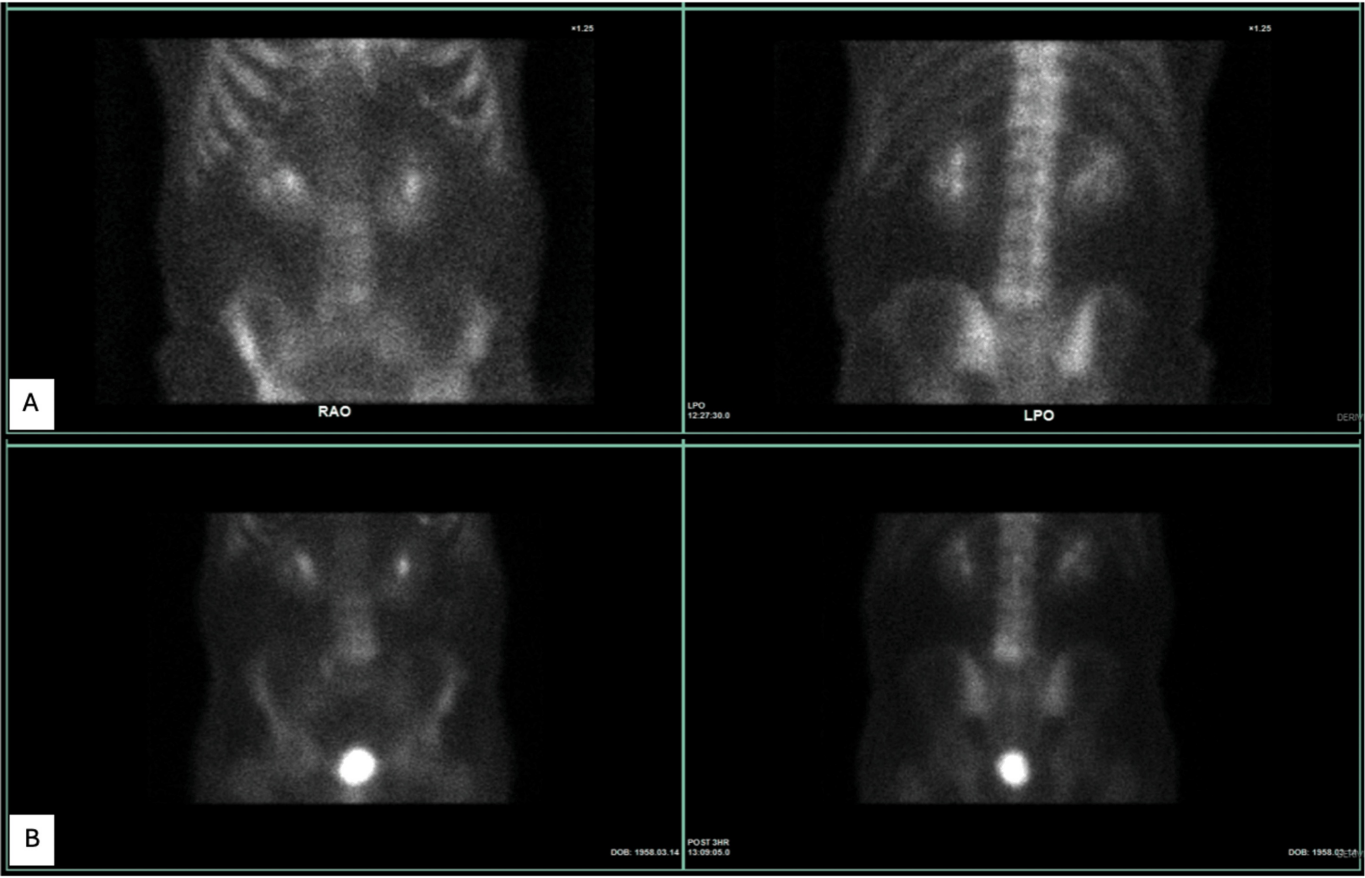
Technetium pyrophosphate nuclear scintigraphy was performed as part of the patient's work-up for the lesion. (A) The first scan performed 32 months after cancer diagnosis, which demonstrated mildly increased uptake in the left side of L5, corresponding to the lesion found on computed tomography in Figure 2. (B) Due to the rather large interval increase in the size of the L5 lesion and the appearance of a new lesion in L4, a repeat scan was performed 47 months after cancer diagnosis, which demonstrated a stable appearance of the mildly increased uptake in L5 identified previously, slightly increased uptake adjacent to this in the inferior margin of L4, and likely non-bridging diffuse idiopathic skeletal hyperostosis in the thoracic spine.

**Figure 4:**
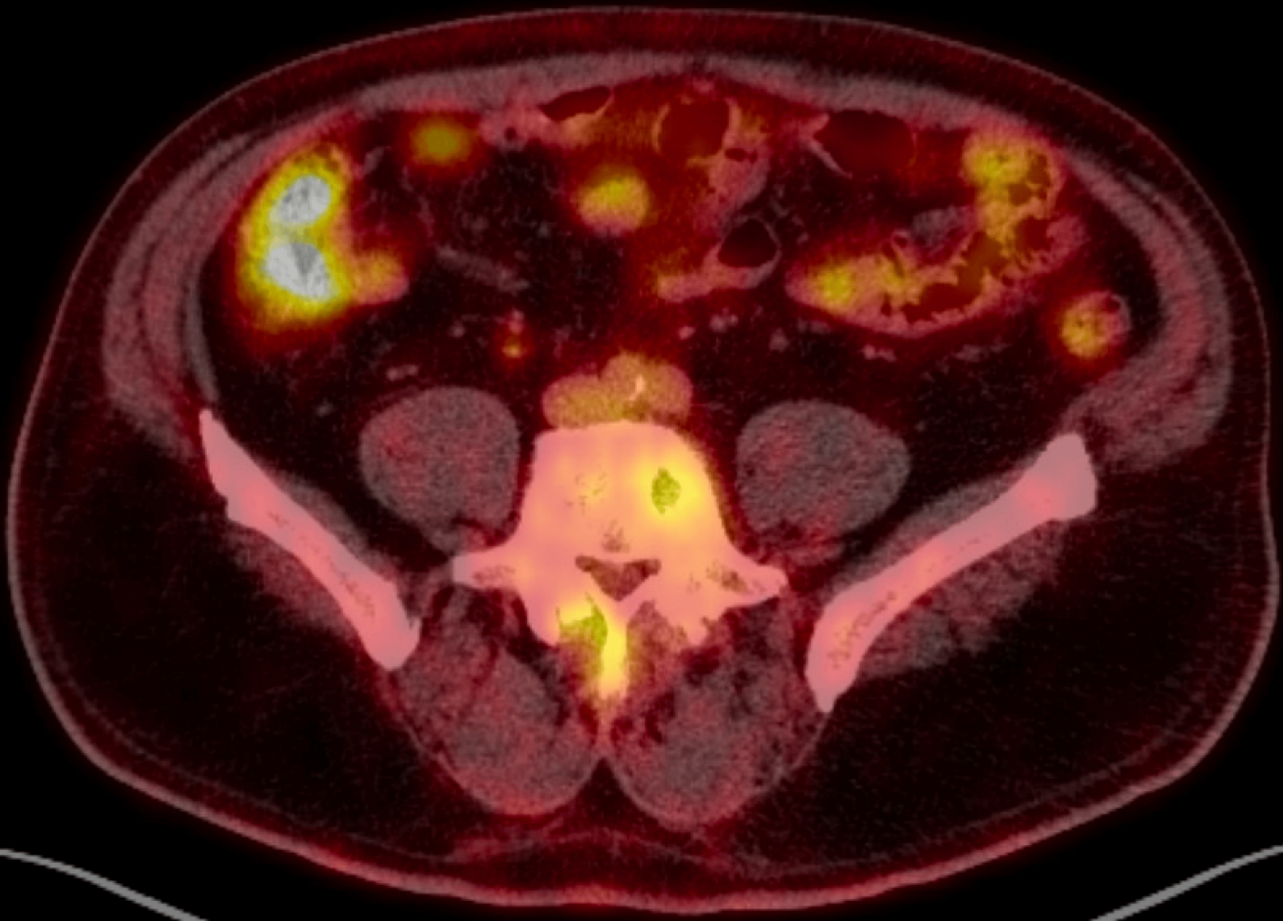
18 fluorine fluorodeoxyglucose positron emission tomography-computed tomography scan was performed 33 months after cancer diagnosis, which demonstrated a mildly increased uptake in the left side of L5, corresponding to the lesion found on computed tomography in Figure 2.

The patient's L5 vertebral body lesion was first discovered on CT of the lumbar spine obtained at an outside facility as part of an evaluation for neurogenic claudication, approximately 2.5 years after his diagnosis of prostate cancer and approximately 1.5 years prior to his presentation to our office. At that time, the hypodense lesion measured 18.7×12.6×12 mm (Figure 2). Technetium pyrophosphate nuclear scintigraphy (bone scan) and 18 fluorine fluorodeoxyglucose (^18^F-FDG) positron emission tomography-CT (PET-CT) scan were performed as part of the patient's subsequent work-up for the lesion, which demonstrated mildly increased uptake in the left side of L5, corresponding to the lesion found on CT (Figure 3A and Figure 4, respectively). A CT-guided fine-needle aspiration (FNA) of the lesion was performed, which demonstrated fibrin, scattered inflammatory cells, and no neoplastic findings. Given the reassuring FNA findings, the patient subsequently underwent an L4-L5 microscopic unilateral laminotomy with bilateral decompression, which provided some relief of his neurogenic symptoms for a period of several months.

**Figure 5:**
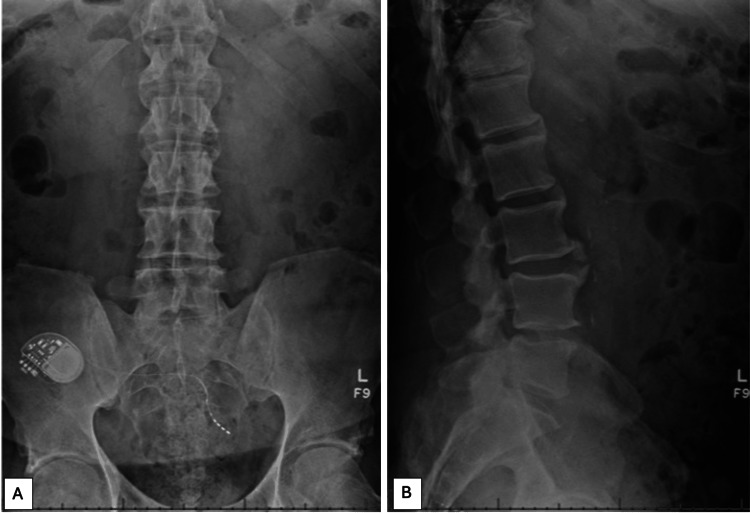
Plain film standing radiographs of the lumbar spine obtained several months after lumbar decompression, as neurogenic claudication symptoms returned. No obvious destructive or expansive osseous lesions were readily identifiable. Figure 5A demonstrates an anterior-to-posterior radiograph of the lumbar spine, while Figure 5B demonstrates a lateral radiograph.

**Figure 6:**
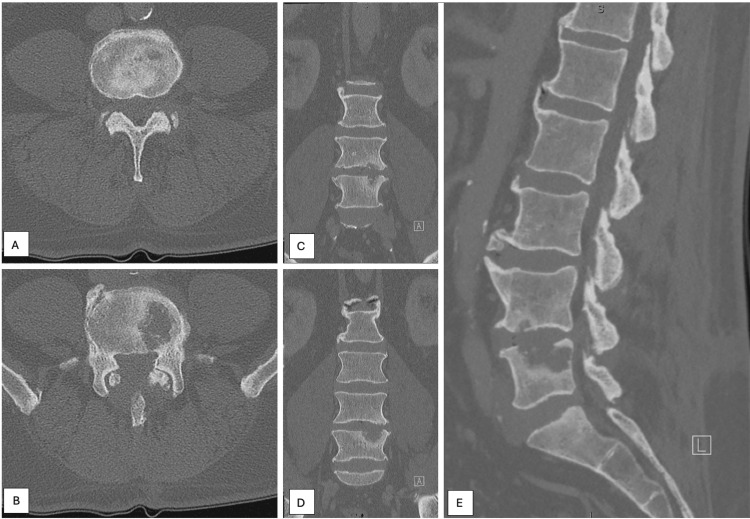
Repeat non-contrast computed tomography of the lumbar spine was performed 47 months after cancer diagnosis and demonstrated a marked interval expansion of the erosive L5 lesion with poorly defined margins, which now measured 30.4×22.6×13.8 mm. In addition, a hypodense, erosive lesion in the left side of L4 was also identified, which measured 4×13.9×4.3 mm. Figure 6A demonstrates an axial cut at the level of the L4 vertebral body lesion, while Figure 6B demonstrates the lesion in the L5 vertebral body. Figures 6C and 6D demonstrate coronal cuts, with Figure 6C showing both lesions and demonstrating a more anterior cut than the image presented in Figure 6D. Figure 6E demonstrates the L4 and L5 vertebral body lesions on a sagittal image.

His neurogenic claudication gradually returned, and he presented to his spine surgeon for further evaluation. Plain film standing radiographs of the lumbar spine were obtained with limited benefit, as no radiolucent lesions within L5 could readily be identified (Figure 5). Repeat CT of the lumbar spine demonstrated marked interval expansion of the erosive L5 lesion with poorly defined margins, which now measured 30.4×22.6×13.8 mm (Figure 6). In addition, a hypodense, erosive lesion in the left side of L4 was also identified, which measured 4×13.9×4.3 mm. Due to the rather large increase in the size of the L5 lesion and the appearance of a new lesion in L4, a repeat bone scan was performed, which demonstrated a stable appearance of the mildly increased uptake in L5 identified previously, slightly increased uptake adjacent to this in the inferior margin of L4, and likely non-bridging diffuse idiopathic skeletal hyperostosis in the thoracic spine (Figure 3B). Given this clear progression of the lesions, the patient underwent a repeat FNA, along with a CT-guided core needle biopsy (CNB) of the lesion and bone marrow biopsy of the left iliac wing at the outside facility. The repeat FNA yielded a non-diagnostic specimen. The CNB of the lesion demonstrated fibrous tissue, benign bone, and no neoplastic findings. The bone marrow aspirate demonstrated no evidence of clonality. Laboratory panels, including PSA and standard markers for multiple myeloma, were all reassuring. The patient was subsequently referred to our cancer institute for further evaluation and treatment recommendations.

It was clear that the patient had undergone an extensive and thorough work-up prior to presenting to our institution (Table 1). It is important to note that magnetic resonance imaging (MRI) was not obtained despite the knowledge that the additional information it could provide could have been valuable. Unfortunately, the patient could not undergo MRI, due to an incompatible bladder stimulator that was previously implanted for his mixed urinary incontinence. Due to the new lesion crossing the L4-L5 disc space and affecting the L4 inferior endplate, we broadened the differential diagnosis to include infection, with a particular focus on a more atypical infection (e.g., fungal), due to the very slow radiographic changes. Out of an abundance of caution, we recommended proceeding with a repeat CT-guided CNB performed by physicians at our institution, to include specimens sent for cultures. A repeat CT-guided CNB of the lesion was subsequently obtained at our institution, which demonstrated benign bone remodeling and fibrocartilage; bacterial and fungal cultures of this specimen demonstrated no growth. Of note, during the course of this more recent work-up, the patient also underwent bilateral L4-L5 epidural corticosteroid injections, which provided good relief of his neurogenic claudication symptoms.

**Table 1:** Chronologic summary of events in the work-up of the patient's lumbar spine lesions, with the approximate amount of time since the patient's prostate cancer was originally diagnosed. Where applicable, values represent the largest axial×coronal×sagittal dimensions. ^a^Bone scan refers specifically to technetium pyrophosphate nuclear scintigraphy. ^b^Laboratory panel included total prostate-specific antigen, thyroid panel, immunofixation electrophoresis, complete blood count with differential, and comprehensive metabolic panel. CT: computed tomography; ^18^F-FDG PET-CT: 18 fluorine fluorodeoxyglucose positron emission tomography-CT; FNA: fine-needle aspiration; CNB: core needle biopsy; BMA: bone marrow aspiration

Diagnostic modality	Elapsed time since cancer diagnosis (April 2019)	Findings
Non-contrast CT of the lumbar spine	31 months	New hypodense L5 lesion measuring 18.7×12.6×12 mm (see Figure 2)
Bone scan^a^	32 months	Mildly increased uptake in the left side of L5 corresponding to the lesion seen on CT (see Figure 3A)
^18^F-FDG PET-CT	33 months	Mildly hypermetabolic L5 lesion corresponding to the lesion seen on CT (see Figure 4)
CT-guided FNA	33.5 months	Fibrin, scattered inflammatory cells, and no neoplastic findings
Non-contrast CT of the lumbar spine	47 months	Marked interval expansion of the hypodense L5 lesion, now measuring 30.4×22.6×13.8 mm; new hypodense L4 lesion measuring 4×13.9×4.3 mm (see Figure 6)
Bone scan^a^	47 months	Increased uptake in the left side of L5, stable from prior exam; mildly increased uptake in the inferior margin of L4, adjacent to the L5 lesion (see Figure 3B)
CT-guided FNA	47.5 months	Non-diagnostic specimen
CT-guided CNB	47.5 months	Fibrous tissue, benign bone, and no neoplastic findings
CT-guided BMA	47.5 months	No evidence of clonality
Laboratory panel^b^	48.5 months	Within normal limits
CT-guided CNB	49 months	Benign bone remodeling and fibrocartilage; bacterial and fungal cultures of this specimen demonstrated no growth
Open biopsy	50 months	Benign-appearing disorganized fibrocartilage, consistent with Schmorl’s node (see Figure 7)

Based on the available imaging, laboratory markers, and pathology findings, the differential diagnosis included an atypical presentation of a degenerative condition (e.g., atypical Schmorl's node), atypical spondylodiscitis (e.g., fungal, aseptic, non-infectious), or malignancy. The patient's pathology and laboratory findings were reassuring against malignancy or pyogenic infection, and his other imaging findings and known history of spinal stenosis with symptomatic improvement following prior decompression and corticosteroid injections favored a degenerative etiology. Further, it was unclear whether his vertebral body lesions were contributing at all to his neurogenic claudication. Notwithstanding, the clear, progressive enlargement and erosive nature of the lesions in the setting of the patient's known history of prostate cancer compelled a more definitive surgical intervention for diagnosis and symptom treatment.

After an extensive discussion with the patient, the decision was ultimately made to proceed with an open biopsy of the L5 lesion with partial L5 corpectomy via left-sided transpedicular approach and L4-S1 decompression and instrumented posterolateral spinal fusion with placement of non-structural interbody graft (morselized local autograft and allogeneic corticocancellous chips). It was explained to the patient that the primary purpose of the operation was to remove material from the lesion, directly visualize it, and have ample tissue for histopathological analysis. It was explained to the patient that the fusion portion of the procedure was necessary because the bony resection for the transpedicular approach and partial corpectomy would render the L4-L5 motion segment unstable. Stabilization across this segment would require instrumentation and fusion from L4 to S1. It was explained that this was likely a more extensive intervention than what may have been recommended for his moderate spinal stenosis but that the procedure may provide some relief for his spinal stenosis symptoms.

**Figure 7:**
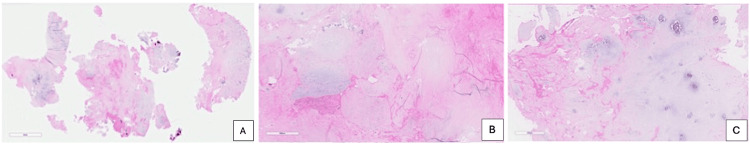
Intraoperative frozen section of a portion of the specimen confirmed the presence of disorganized, typical-appearing fibrocartilage, consistent with an intraspongious/intravertebral herniated nucleus pulposus (Schmorl's node) from the L4 to L5 level. All sections were hematoxylin and eosin stained. The magnifications are 4× (A), 10× (B), and 20× (C).

**Figure 8:**
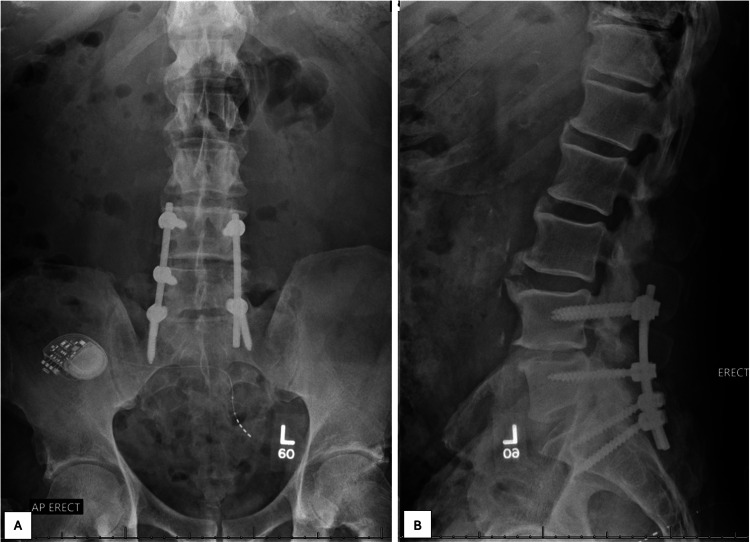
Plain film standing radiographs of the lumbar spine obtained two months postoperatively, which demonstrated no concerning osseous findings, good alignment, and stable appearance of posterior instrumentation. Figure 8A demonstrates an anterior-to-posterior radiograph of the lumbar spine, while Figure 8B demonstrates a lateral radiograph of the lumbar spine. The patient also reported resolution of his back and leg pain at this point.

As part of the informed consent, we explained to the patient our contingency plans for the operation, should our gross findings and/or intraoperative pathology demonstrate infection or neoplastic disease (e.g., more aggressive decompression/debridement, possible local antibiotic placement, etc.). In order to reduce any anatomic confusion and assist with the localization of the lesions, we utilized intraoperative CT with stereotactic navigation (Medtronic O-Arm intraoperative imaging and Medtronic StealthStation surgical navigation; Medtronic, Minneapolis, Minnesota, United States). Intraoperatively, the material removed from the L5 lesion was noted to be dry and non-purulent-appearing and had the gross appearance of disc material. Frozen section of a portion of the specimen confirmed the presence of typical-appearing fibrocartilaginous disc material (Figure 7). Based on these intraoperative findings and subsequent final histopathologic evaluation, the lesion was definitively diagnosed as a large, aggressive, intraspongious/intravertebral herniated nucleus pulposus from the L4 to L5 level. The patient was able to be discharged home on postoperative day 1. Two months postoperatively, imaging demonstrated no concerning findings, and the patient reported resolution of his back and leg pain (Figure 8). 

## Discussion

Prostate cancer is one the most common cancers to spread to the bone [1]. The spinal column is the most common site for osseous metastasis, and over one-half of patients with prostate cancer may develop metastatic spine disease [2]. Prostate cancer may produce sclerotic- or lytic-appearing bone lesions, though lytic is far less common [3-5]. It is crucial to maintain a very high index of suspicion for metastatic disease in adult patients who present with a new lytic-appearing bone lesion, especially those with a known history of malignancy with a high predilection for osseous metastasis [6].

Skeletal biopsy is often required for the definitive diagnosis or verification of a concerning spinal lesion [6]. While diagnostic accuracy (e.g., establishment of diagnosis) rates, particularly for lytic-appearing bone lesions, approach 90-95% with modern biopsy techniques, the true validity of a negative or benign histopathological diagnosis is not entirely understood [1,4,12-15]. A negative tissue sample may reflect a truly benign condition, but may also be a false negative (e.g., sample captured benign tissue immediately adjacent to the tumor, etc.) or non-diagnostic (e.g., inadequate sample, crushed tissue, etc.) [14]. Open biopsy is generally considered to have higher diagnostic accuracy than percutaneous transpedicular biopsy, due to the presumption that more tissue can be obtained via an open technique [12]. On the other hand, modern CT-guided CNB still often provides adequate tissue, and there is limited evidence showing no significant difference in diagnostic accuracy between open and CT-guided biopsy of lytic spinal lesions [16]. The two techniques offer different benefits and carry different risk profiles [16].

In this case report, a 65-year-old male with a history of prostate cancer presented with enlarging, destructive-appearing lesions in the vertebral bodies of L4 and L5, raising concern for new oligometastatic spine disease. An appropriate and thorough initial work-up was performed, which seemed to support a benign, degenerative etiology. In addition, the favorable histopathology upon initial cancer diagnosis and stable surveillance PSA tests following prostatectomy both favored against new, recurrent, or distant disease. However, as discussed, FNA and CNB techniques have limitations, and certain concerning features prompted additional work-up. While it has been reported that benign Schmorl's nodes can indeed have FDG uptake on ^18^F-FDG PET-CT scans, there are no reported normal limits in the avidity of uptake, leading to difficulty differentiating Schmorl's nodes from metastasis in this setting [17,18]. Although MRI may have potentially been able to better characterize the features and internal consistency of the lesion, as has been done in similar clinical scenarios, it is also a possibility that the additional imaging would have further obfuscated the clinical picture and that open biopsy would still be necessary [19].

It has been previously established that GG1 prostate cancer is generally indolent, with some clinicians even favoring active surveillance over prostatectomy [20]. Further, for organ-confined GG1 T2 lesions such as the current patient had, the overall risk of biochemical recurrence after prostatectomy is less than 1.5% [20]. However, it has been previously noted that the cells of GG1 can look morphologically indistinguishable from those of higher-grade cancers, which carry a higher risk of recurrence and metastasis [20]. Given the presence of a number of reassuring features (normal laboratory values, benign CNB) as well as certain more concerning features (interval lesion expansion and erosive nature) of the patient's presentation, a thorough discussion with the patient regarding pathways forward was crucial. Shared decision-making as well as an emphasis on the patient's understanding of the purpose and utility of surgery in his particular situation was a key part of the decision to proceed forward with surgery.

## Conclusions

Although the pretest probability of new metastatic disease in the current patient was low based on all of the available evidence, a myriad of factors introduced a level of uncertainty in this case and compelled more invasive intervention. Namely, the radiographic progression of a lytic lesion with poor margination prompted a more definitive intervention. Differentiation of non-neoplastic conditions, such as a Schmorl's node, from osseous metastatic disease is essential for the appropriate management of patients with a history of malignancy. This case illustrates that, in the event a patient's history, physical examination, imaging, and/or other findings are discordant or in any other way worrisome, further work-up with imaging, laboratory studies, and in certain cases surgical intervention should be undertaken to rule out malignancy. Patients with concerning osseous lesions of the spine should be referred to a specialized cancer treatment center equipped with the expertise and resources to accurately evaluate, diagnose, and manage these lesions.

## Appendices

**Table 2:** Differential diagnosis of non-neoplastic conditions that can mimic metastatic extradural spine disease, as well as benign and malignant neoplastic spine conditions. POEMS: polyneuropathy, organomegaly, endocrinopathy, monoclonal plasma cell disorder, skin changes

Category	Examples
Non-neoplastic conditions ("mimickers")	
Anatomic variants	Red (hematopoietic) marrow hyperplasia, normal heterogeneous yellow (fatty) marrow variant
Trauma	Benign compression fracture (e.g., osteoporotic wedging), reactive (e.g., fracture hematoma/callus)
Degenerative	Discogenic sclerosis (e.g., Modic endplate changes), intraspongious/intravertebral herniated nucleus pulposus (Schmorl's node), Baastrup disease, Charcot spinal arthropathy
Infection	Vertebral osteomyelitis/spondylitis and discitis/spondylodiscitis
Metabolic	Paget's disease of the bone, brown tumor of hyperparathyroidism, fibrous dysplasia, osteopetrosis, renal osteodystrophy, Gaucher's disease
Inflammatory	Certain conditions in other categories could also be classified here
Congenital/developmental	Certain conditions in other categories could also be classified here
Vascular	Post-traumatic avascular necrosis (Kummell disease)
Iatrogenic	Post-radiation/post-chemotherapy changes (e.g., osteolytic metastases that "heal" and become sclerotic with treatment), osteolysis (e.g., bone morphogenetic protein-2, periprosthetic metallosis, etc.), bone cement (e.g., vertebroplasty/kyphoplasty)
Rare ("zebras")	Auto-osteolysis (e.g., Gorham-Stout disease, Hajdu-Cheney disease, etc.)
Neoplastic conditions	
Benign	Benign notochordal cell tumor
	Vertebral hemangioma
	Giant cell tumor/osteoclastoma
	Osteoid osteoma/osteoblastoma
	Osteochondroma/chondroma/osteocartilaginous exostosis
	Eosinophilic granuloma
	Bone cyst (e.g., unicameral bone cyst, intraosseous ganglion, aneurysmal bone cyst including giant cell (reparative) granuloma (solid variant of aneurysmal bone cyst))
	Other chondroid lesions (e.g., enchondroma, chondroblastoma)
	Other fibrous lesions (e.g., non-ossifying fibroma, fibrous dysplasia, chondromyxoid fibroma)
	Vascular conditions (e.g., angiolipoma, hemangioendothelioma (e.g., Masson's vegetant intravascular hemangioendothelioma, pseudomyogenic hemangioendothelioma, etc.))
	Intraosseous lipoma
	Langerhans cell histiocytosis
Malignant (primary)	Chordoma
	Osteosarcoma (osteogenic sarcoma)
	Primary Ewing sarcoma
	Primary lymphoma
	Chondrosarcoma
	Multiple myeloma/solitary plasmacytoma (some would not classify this as a primary bone tumor)
	Neuroblastic/neuroectodermal tumors (e.g., ganglioneuroma, ganglioneuroblastoma, neuroblastoma)
	Hemangiopericytoma
	Non-osteogenic, non-Ewing soft tissue sarcoma of the bone (e.g., fibrosarcoma (e.g., dermatofibrosarcoma protuberans), angiosarcoma, leiomyosarcoma, rhabdomyosarcoma, undifferentiated pleomorphic sarcoma of bone (malignant fibrous histiocytoma), alveolar soft part sarcoma, malignant peripheral nerve sheath tumor, etc.)
Malignant (secondary/metastatic and hematological)	Metastatic tumors (e.g., lung, breast, prostate, renal, etc.)
	Secondary lymphoma
	Multiple myeloma (including POEMS/polyneuropathy, organomegaly, endocrinopathy, monoclonal plasma cell disorder, skin changes)
	Solitary plasmacytoma
	Focal infiltration of leukemia (including chloroma/granulocytic sarcoma/extramedullary myeloblastoma)
	Myelofibrosis
	Metastatic myxoid liposarcoma
	Metastatic Ewing sarcoma

## References

[1] Dodwad SN, Savage J, Scharschmidt TJ, Patel A (2014). Evaluation and treatment of spinal metastatic disease. Orthopaedic Oncology. Cancer Treatment and Research.

[2] Moussazadeh N, Laufer I, Yamada Y, Bilsky MH (2014). Separation surgery for spinal metastases: effect of spinal radiosurgery on surgical treatment goals. Cancer Control.

[3] Creek AT, Ratner DA, Porter SE (2014). Evaluation and treatment of extremity metastatic disease. Orthopaedic Oncology. Cancer Treatment and Research.

[4] Sciubba DM, Petteys RJ, Dekutoski MB (2010). Diagnosis and management of metastatic spine disease. A review. J Neurosurg Spine.

[5] Segamwenge IL, Mgori NK, Abdallahyussuf S, Mukulu CN, Nakangombe P, Ngalyuka PK, Kidaaga F (2012). Cancer of the prostate presenting with diffuse osteolytic metastatic bone lesions: a case report. J Med Case Rep.

[6] Rougraff BT, Kneisl JS, Simon MA (1993). Skeletal metastases of unknown origin. A prospective study of a diagnostic strategy. J Bone Joint Surg Am.

[7] Gebauer GP, Farjoodi P, Sciubba DM, Gokaslan ZL, Riley LH 3rd, Wasserman BA, Khanna AJ (2008). Magnetic resonance imaging of spine tumors: classification, differential diagnosis, and spectrum of disease. J Bone Joint Surg Am.

[8] Ross JS, Moore KR (2021). Neoplasms, cysts, and other masses. Diagnostic Imaging: Spine.

[9] Compagnone D, Cecchinato R, Pezzi A (2023). Diagnostic approach and differences between spinal infections and tumors. Diagnostics (Basel).

[10] Abdel Razek AA, Castillo M (2010). Imaging appearance of primary bony tumors and pseudo-tumors of the spine. J Neuroradiol.

[11] Macedo F, Ladeira K, Pinho F, Saraiva N, Bonito N, Pinto L, Goncalves F (2017). Bone metastases: an overview. Oncol Rev.

[12] Avedian RS (2014). Principles of musculoskeletal biopsy. Orthopaedic Oncology. Cancer Treatment and Research.

[13] Dave BR, Nanda A, Anandjiwala JV (2009). Transpedicular percutaneous biopsy of vertebral body lesions: a series of 71 cases. Spinal Cord.

[14] Lange MB, Petersen LJ, Nielsen MB, Zacho HD (2021). Validity of negative bone biopsy in suspicious bone lesions. Acta Radiol Open.

[15] Rimondi E, Staals EL, Errani C (2008). Percutaneous CT-guided biopsy of the spine: results of 430 biopsies. Eur Spine J.

[16] Yapici F, Atici Y, Balioglu MB (2015). A comparison of two techniques: open and percutaneous biopsies of thoracolumbar vertebral body lesions. J Craniovertebr Junction Spine.

[17] Daignault CP, Palmer EL, Scott JA, Swan JS, Daniels GH (2015). Papillary thyroid carcinoma metastasis to the lumbar spine masquerading as a Schmorl's node. Nucl Med Mol Imaging.

[18] Oh E, Kim HJ, Kwon JW, Yoon YC, Kim HS (2022). Differentiation between spinal subchondral bone metastasis with focal pathologic endplate fracture and oedematous Schmorl's node. J Med Imaging Radiat Oncol.

[19] Niwa N, Nishiyama T, Ozu C, Yagi Y, Saito S (2015). Schmorl nodes mimicking osteolytic bone metastases. Urology.

[20] Epstein JI (2022). Is grade group 1 (Gleason score 3 + 3 = 6) adenocarcinoma of the prostate really cancer?. Curr Opin Urol.

